# C6 peptide blockade of Hv1 channels inhibits neutrophil migration into the lungs to suppress *Pseudomonas aeruginosa*-induced acute lung injury

**DOI:** 10.1186/s12931-025-03409-0

**Published:** 2025-11-28

**Authors:** Ruiming Zhao, Benjamin Lopez, Nairrita Majumder, Aren Kasparian, Punyanuch Hua, Hui Dai, Maha Nayak, Tatiana Zyrianova, Clove Taylor, Yiwen Ding, Colin J. Sallee, Anil Sapru, Steve A.N. Goldstein, Andreas Schwingshackl

**Affiliations:** 1 Departments of Pediatrics and Physiology & Biophysics, School of Medicine, Department of Pharmaceutical Sciences, School of Pharmacy and Pharmaceutical Sciences, Susan and Henry Samueli College of Health Sciences, University of California, Irvine, CA 92697 USA; 2 Department of Pediatrics, University of California Los Angeles, Los Angeles, CA 90095 USA

**Keywords:** Acute lung injury (ALI), Neutrophils, RNA sequencing (RNA-seq), Voltage-gated proton channels.

## Abstract

**Background:**

Acute Lung Injury (ALI) and its most severe form, Acute Respiratory Distress Syndrome (ARDS), are critical pulmonary conditions characterized by life-threatening acute hypoxic respiratory failure, affecting over three million individuals globally each year. ALI involves alveolar inflammation and disruption of the alveolar-capillary barrier, primarily driven by neutrophil infiltration and the release of inflammatory mediators. In our previous study using a lipopolysaccharide (LPS)-induced mouse model of ALI, we demonstrated that C6, a peptide inhibitor of voltage-gated proton channels (Hv1), ameliorates lung injury, identifying Hv1 as a potential therapeutic target. However, (i) whether the anti-inflammatory effects of C6 are translatable to a clinically relevant live bacterial infection model, and (ii) the molecular mechanisms underlying these anti-inflammatory effects, remain unknown, and are a crucial next step towards targeted rational drug development.

**Methods:**

To induce ALI, we used an intratracheal *Pseudomonas aeruginosa* infection model, a gram-negative bacterium relevant in ventilated and immunocompromised patients. A separate group of infected mice also received intravenous treatment with C6 (4 mg/kg). Lung injury severity was evaluated using histopathological analysis. Bronchoalveolar lavage (BAL) fluid was collected to quantify neutrophil infiltration and proinflammatory cytokines concentrations. In addition, reactive oxygen species (ROS) production and intracellular calcium levels in BAL neutrophils were measured. RNA sequencing of BAL neutrophils was conducted to assess C6-induced transcriptional changes. Key findings were validated in vitro using human neutrophils.

**Results:**

C6 mitigates P. aeruginosa-induced ALI in mice by reducing neutrophil infiltration into the alveolar space by ~ 86%, improving lung injury scores, decreasing BAL fluid proinflammatory cytokine levels, and suppressing neutrophil ROS production and intracellular calcium levels. RNA sequencing of BAL neutrophils revealed 51 downregulated genes, including key regulators of neutrophil migration, cytokine release, and ROS production; only three genes were upregulated and they also have roles in neutrophil immune defense. In human neutrophils, C6 similarly inhibited chemotaxis and reduced ROS and cytokine release, and calcium influx.

**Conclusions:**

Targeting Hv1 with C6 effectively protects against P. aeruginosa-induced ALI by limiting neutrophil recruitment and activation. These findings establish C6 as a promising therapeutic candidate against infectious ALI and provide important mechanistic insights into its immunomodulatory effects on neutrophils.

**Supplementary Information:**

The online version contains supplementary material available at 10.1186/s12931-025-03409-0.

## Background

Infectious pneumonia is the leading cause of hospital admissions in the United States [1]. *Pseudomonas aeruginosa* (P. aeruginosa), a Gram-negative, aerobic, rod-shaped bacterium, is the leading species isolated from patients with nosocomial infection and is associated with particularly poor patient outcomes, in part due to increasing antibiotic resistance patterns, highlighting the need for new therapeutic approaches [2]. Acute lung injury (ALI), including its most severe manifestation, Acute Respiratory Distress Syndrome (ARDS), represent a devastating clinical condition responsible for more than 190,000 hospitalizations and approximately 74,500 deaths annually in the United States. The overall mortality rate remains around 40% [3, 4]. Beyond antibiotic therapy, no molecular targets have yet been identified that translate into improved patient outcomes, and current management remains largely supportive [5].

Neutrophils are key effector cells of the innate immune system against bacterial lung infections [6]. They are chemotactically recruited from the bloodstream to the alveolar spaces where they play a critical role in bacterial killing [6], but as part of the immune response, they are also responsible for disrupting the alveolar-capillary barrier and damaging the lung parenchyma by producing and releasing reactive oxygen species (ROS), proteases, and cytokines, allowing protein-rich fluid to accumulate in the alveoli, further impeding gas exchange [6, 7].

Because the voltage-gated proton channel (Hv1) is highly expressed on neutrophils (6–9), we recently explored its potential role as a therapeutic target using a lipopolysaccharide (LPS)-induced lung injury model for proof of concept [8]. LPS, an endotoxin derived from the outer membrane of Gram-negative bacteria, is non-replicative and incapable of causing a sustained infection. Unlike bacterial infections, which evolve over time and involve complex host-pathogen interactions, LPS-induced lung injury is acute, self-limiting, and lacks the dynamic pathophysiological features observed in clinical ALI. Although LPS exposure triggers neutrophilic inflammation and cytokine release, depending on the administered dose, the resultant lung injury is either lethal or resolves relatively quickly within 24 to 72 h.

To investigate Hv1 channel function, we developed C6, the first specific, high-affinity peptide inhibitor of Hv1 [9]. To generate C6, we constructed a combinatorial library of approximately one million novel peptides based on the inhibitor cystine knot (ICK) scaffold of gating-modifier toxins, which bind to the voltage-sensing domain of voltage-gated ion channels. These peptides interact with the voltage sensors to modulate their movement and display state-dependent binding affinities [10]. Phage clones expressing C6 were selected for their ability to bind purified Hv1 channels. C6 is a 41-amino acid peptide whose ICK structure is stabilized by three disulfide bonds. The synthesized peptide inhibits Hv1 in a dose-dependent, voltage-dependent, and cooperative manner, with an inhibition constant (*K*_i_) of 1.5 nM [9, 11], as determined in HEK293T cells expressing Hv1 channels using whole-cell patch-clamp recordings at 0 mV. Notably, our synthetic peptide inhibitor, C6, is unrelated to the complement system component of the same name.

In the sublethal LPS model used in our proof of concept study, C6 peptide demonstrated promising anti-inflammatory effects [8]. However, the transient and self-resolving proinflammatory effect of LPS does not adequately replicate the persistent and progressive nature of ALI associated with live bacterial etiologies in human pneumonia [12]. To overcome these crucial limitations in the current study, we designed a pneumonia model using intratracheal (i.t.) instillation of live P. aeruginosa bacteria. We demonstrate that C6 confers protection in this clinically relevant model by suppressing the migration of circulating neutrophils into the lungs by ~ 86%. Mechanistically, C6-mediated inhibition of neutrophil recruitment is associated with suppressed alveolar levels of proinflammatory cytokines and chemokines, as well as reductions in ROS and intracellular Ca^2+^ levels of neutrophils. Furthermore, RNA sequencing revealed downregulation of six genes involved in neutrophil migration, cytokine production, and ROS generation following C6 treatment. These protective mechanisms observed in mice were also replicated in human neutrophils.

## Methods

### Animals

All experiments were conducted using WT C57BL/6 mice aged 9–12 weeks and purchased from The Jackson Laboratories. For experimental purposes, mice were age- and sex-matched as closely as possible. Approval for experiments in mice was obtained from the University of California Los Angeles Animal Research Committee (ARC). All experiments were performed in accordance with institutional protocols, guidelines, and recommendations, as well as the ARRIVE 2.0 guidelines.

### Peptide synthesis and purification

C6 peptide (GenBank: AZI15804) and KTx* peptides were synthesized by CSBio. Peptide folding reactions were quenched by acidification and the peptides were purified by HPLC, as before [9]. Peptides that were >90% pure were lyophilized and stored at − 20 °C. Peptides were dissolved in solutions for in vitro or in vivo experiments before use.

### P. aeruginosa-induced acute lung injury and C6 peptide administration

Mice were anesthetized with brief inhalation of isofluorane (2–5%) until they lost consciousness, then were suspended by their incisors on a 3.0 silk suture mounted on a 45 degree-angled stand. The tongue was gently extracted from the mouth and moved to the side using blunt forceps to visualize the vocal cords. Using fiberoptic guidance, a 20-gauge angiocatheter was passed through the vocal cords into the subglottic area, and 40 µL of P. aeruginosa (2 × 10^7^ CFU) or PBS vehicle control were injected with a micropipettor. We chose 2 × 10^7^ CFU of P. aeruginosa based on our pilot time- and dose-response studies. This dose at the 72-hour time point provided us with clinically-relevant model of moderate-severe acute lung injury with the opportunity for meaningful therapeutic intervention. Mice were then placed back into their native cages and allowed to recover until fully awake. No perianesthetic deaths were associated with this procedure. C6 stock solution was prepared in a mixture of DMSO and PBS (DMSO: PBS = 1:20). 4 mg/kg C6 was used based on our previous study [8]. Five hours after i.t. P. aeruginosa infection, the first dose of C6 (100 µL) was injected intravenously (i.v.) via the retro-orbital vein. At 24 and 48 h after P. aeruginosa infection, two more doses of C6 were injected. This once-daily drug regimen is a clinically-feasible approach. Vehicle controls for C6 contained DMSO and PBS (DMSO: PBS = 1:20).

### Lung histology, BAL fluid collection, neutrophil counts, IgM, ROS, and [Ca^2+^]_in_ quantification

After 72 h of P. aeruginosa infection, a tracheostomy was performed using an 18-gauge steel catheter under general ketamine/xylazine anesthesia (intraperitoneal injection, 100 mg/kg ketamine, 12.5 mg/kg xylazine). BAL fluid was collected from all mice using a 1 mL syringe attached to the tracheostomy catheter. Two washouts were performed with 1 mL PBS for BAL IgM and total cell count determination, and 1 mL PBS/1% BSA for cytokine assays. All samples were immediately placed on ice. IgM concentration was measured using Invitrogen IgM Mouse Uncoated ELISA, and BAL fluid neutrophil counts were performed on cytospins using a Diff-Quick stain (Fisher Scientific). ROS production in BAL fluid cells was measured using Carboxy-H2DCFDA with Cellular ROS Assay (ThermoFisher), and [Ca^2+^]_in_ was determined using a Fluo-4 NW Calcium Assay Kit (Molecular Probes). Following BAL fluid collection, mice were euthanized via cardiac puncture and lungs were removed for histological examination. Briefly, the lungs were gently retrograde perfused via the right ventricle with 10 mL ice-cold PBS to remove red blood cells. Lung tissue was then removed en bloc and immediately perfused and fixed in 4% formalin. Paraffin embedded sections were cut into 4 μm thick tissue slices using a Microtome, and H&E-stained for histology. Two sets of slides were prepared and analyzed for each mouse. Lung injury scores were determined by an investigator blinded to the experimental conditions on H&E-stained lung sections as previously described [13], using the following 3 criteria: (i) interstitial edema, (ii) cellular infiltrate, and (iii) parenchymal, peribronchial and perivascular hemorrhage. Each criterion was assigned a score between 0 and 3, with “0” representing no injury, “1” representing a mild injury, “2” representing a moderate injury, and “3” representing a severe injury. Four randomly assigned high power fields per slide were scored under 20x magnification on a Motic AE20/21 inverted microscope, and scores were averaged for each criterion. Using the sum of these averages, a composite histological lung injury score was calculated for each experimental group.

### Cytokine measurements by luminex’s xMAP^®^ immunoassay

Mouse BAL fluid was centrifuged at 1000 g for 10 min at 4 °C for cytokine/chemokine profiling. Samples were measured and analyzed by the UCLA Immune Assessment Core using a MILLIPLEX MAP Mouse Cytokine/Chemokine Magnetic Bead Panel (Millipore Sigma MCYTMAG-70 K-PX32) on a Luminex 200 instrument (ThermoFisher) as per manufacturer’s instructions. Standard curves were provided by the Core facility.

### Confirmatory cytokine measurements by ELISA

Cytokine concentrations were quantified in BAL fluid after centrifugation at 1000 g for 10 min at 4 °C. Briefly, 100 µL of sample was loaded into 96-well species specific ELISA plates and analyzed in triplicate following the manufacturer’s instructions. Values are displayed in pg/mL.

### RNA-seq analysis

BAL fluid was collected from P. aeruginosa-infected mice, with or without C6 treatment, as described above. BAL neutrophils were isolated with a purity of > 98% using negative magnetic bead selection with the EasySep™ Mouse Neutrophil Enrichment Kit (Stemcell Technologies). Total RNA was extracted using the Qiagen RNeasy Micro Kit in accordance with the manufacturer’s instructions. RNA quantity and integrity were assessed using a NanoDrop 8000 spectrophotometer (Thermo Fisher Scientific) and a 2200 TapeStation system (Agilent Technologies), respectively. A total of 250 ng of RNA was obtained from each group of mice: those infected with P. aeruginosa alone and those treated with C6 post-infection. RNA libraries were prepared using the KAPA mRNA HyperPrep Kit following the manufacturer’s protocol. Briefly, RNA samples underwent poly-A selection, enzymatic fragmentation, and synthesis of double-stranded cDNA using a combination of oligo(dT) and random primers. The resulting cDNA was subjected to end repair, dual-index adaptor ligation, strand selection, and amplification by 15 cycles of PCR. Libraries were purified using the BioChain AnaPrep Automated Nucleic Acid Preparation System and subsequently sequenced on an Illumina NovaSeq X Plus platform at the UCLA Technology Center for Genomics & Bioinformatics (TCGB). Sequencing reads were mapped to the mouse reference genome (GRCm38) and quantified using Salmon v1.10.0. Genes with low expression (defined as fewer than 10 counts in at least five samples) were excluded from downstream analyses. Differential gene expression was evaluated using negative binomial generalized linear models (NB-GLM) as implemented in DESeq2. Genes with an absolute log2 fold change > 0.5 and a false discovery rate (FDR)-adjusted p-value < 0.1 were considered significantly differentially expressed. Enrichment analysis of these genes was conducted using the Database for Annotation, Visualization, and Integrated Discovery (DAVID), with significance defined as FDR < 0.05.

### Isolation of human peripheral blood neutrophils

Human polymorphonuclear neutrophils were isolated from peripheral blood obtained from healthy donors through Ficoll-Paque Plus (GE Healthcare) density-gradient centrifugation. Blood samples were provided by the Institute for Clinical and Translational Science at the University of California, Irvine, and all procedures were approved by the University of California, Irvine’s Institutional Review Board. The donor population comprised an equal distribution of females and males, with ages ranging from 25 to 62 years. A volume of 20 mL of peripheral blood was mixed with 3% dextran in PBS (Sigma-Aldrich) and incubated for 20 min in a 50 mL conical tube to sediment erythrocytes. The upper clear layer containing leukocytes was carefully collected and underlaid with 10 mL of Ficoll-Paque Plus. The mixture was centrifuged at 500 × g for 30 min at 20 °C to separate mononuclear cells from neutrophils and residual red blood cells. The plasma and mononuclear cell layers were aspirated, and the pellet containing neutrophils and red blood cells was resuspended in Red Blood Cell Lysis Buffer (eBioscience) and incubated for 10 min to lyse red blood cells. Lysis was stopped by adding 35 mL of PBS, followed by centrifugation at 300 × g for 5 min at 4 °C. The resulting cell pellet was resuspended in RPMI 1640 medium (Gibco). An aliquot of the neutrophil suspension was mixed with Trypan Blue (Gibco) and counted using a hemocytometer to assess cell viability. This isolation protocol consistently yielded a cell population composed of more than 97% neutrophils.

### Neutrophil migration assay

Human neutrophils were isolated from peripheral blood and resuspended in serum- and phenol red-free RPMI 1640 medium (Gibco). Neutrophil migration was determined using CytoSelect™ 96-Well Cell Migration Assay (3 μm, Fluorometric Format) (Cell Biolabs). C6 and control blockers were added directly to the cell suspension. Neutrophils treated with or without blockers (C6, KTx*, or Zn^2+^) were added to the upper chamber, and known neutrophil-activating agents (25 µg/mL LPS or 0.5 µg/mL IL-1β) were added to the bottom chamber. We employed a single C6 dose (2 µM) in the in vitro studies, based on prior evidence that higher concentrations of C6 were not more effective blocking Hv1 channels in neutrophils or protecting mice from LPS-induced ALI [8, 9, 14]. After 24 h, the neutrophils in the bottom chamber were harvested and quantified using CyQuant GR dye and Fluoroskan FL (ThermoFisher) plate reader equipped with internal software SkanIt 6.02.

### Neutrophil ROS measurement

Human neutrophils were isolated from peripheral blood and resuspended in Hank’s Balanced Salt Solution (HBSS) comprising 138 mM NaCl, 5.4 mM KCl, 0.34 mM Na_2_HPO_4_, 0.44 mM KH_2_PO_4_, 1.3 mM CaCl_2_, 0.5 mM MgCl_2_, 0.4 mM MgSO_4_, 4.2 mM NaHCO_3_, 5.5 mM glucose and 20 mM HEPES, pH 7.2, and dispensed into white 96-Well Immuno Plates (Thermo Fisher). Neutrophil ROS release was measured using Luminol (Sigma) as described [14]. For the C6 group, neutrophils were pre-incubated with 2 µM C6 for 1 h before the assay. After incubation, neutrophils were stimulated with P. aeruginosa and fluorescence was measured immediately using Fluoroskan FL.

### Neutrophil [Ca^2+^]_in_ measurement

Human neutrophils were isolated from peripheral blood and resuspended in HBSS. Neutrophils were incubated with Fluo-3 (5 µM) for 30 min. Cells were washed with HBSS once after incubation and then resuspended in HBSS to allow for complete de-esterification of intracellular Fluo-3 AM esters for an additional 30 min. 2 µM C6 was added during the final incubation. After incubation, neutrophils were stimulated with P. aeruginosa, and fluorescence was measured immediately using Fluoroskan FL.

### Statistics

Data were analyzed using the unpaired Student t-test (two-sided) and one-way ANOVA for multiple comparisons. All statistical analyses were performed using GraphPad Prism 6 software. *p* ≤ 0.05 values were considered significant.

## Results

### Hv1 blockade with C6 peptide suppresses acute lung injury in P. aeruginosa-infected mice

Given the absence of a specific inhibitor of Hv1 we developed the C6 peptide using a high-throughput phage-display strategy and demonstrated that it inhibits human Hv1 [9] and mouse Hv1 [8] with low nM affinity and a high degree of discrimination [9].

In mice, i.t. instillation of P.aeruginosa bacteria (2 × 10^7^ CFU) produces lung injury and inflammation that reproduces many of the findings observed in human ALI [15], including migration of neutrophils into the alveolar air spaces, direct alveolar epithelial damage, and pulmonary edema [16].

**Fig. 1:**
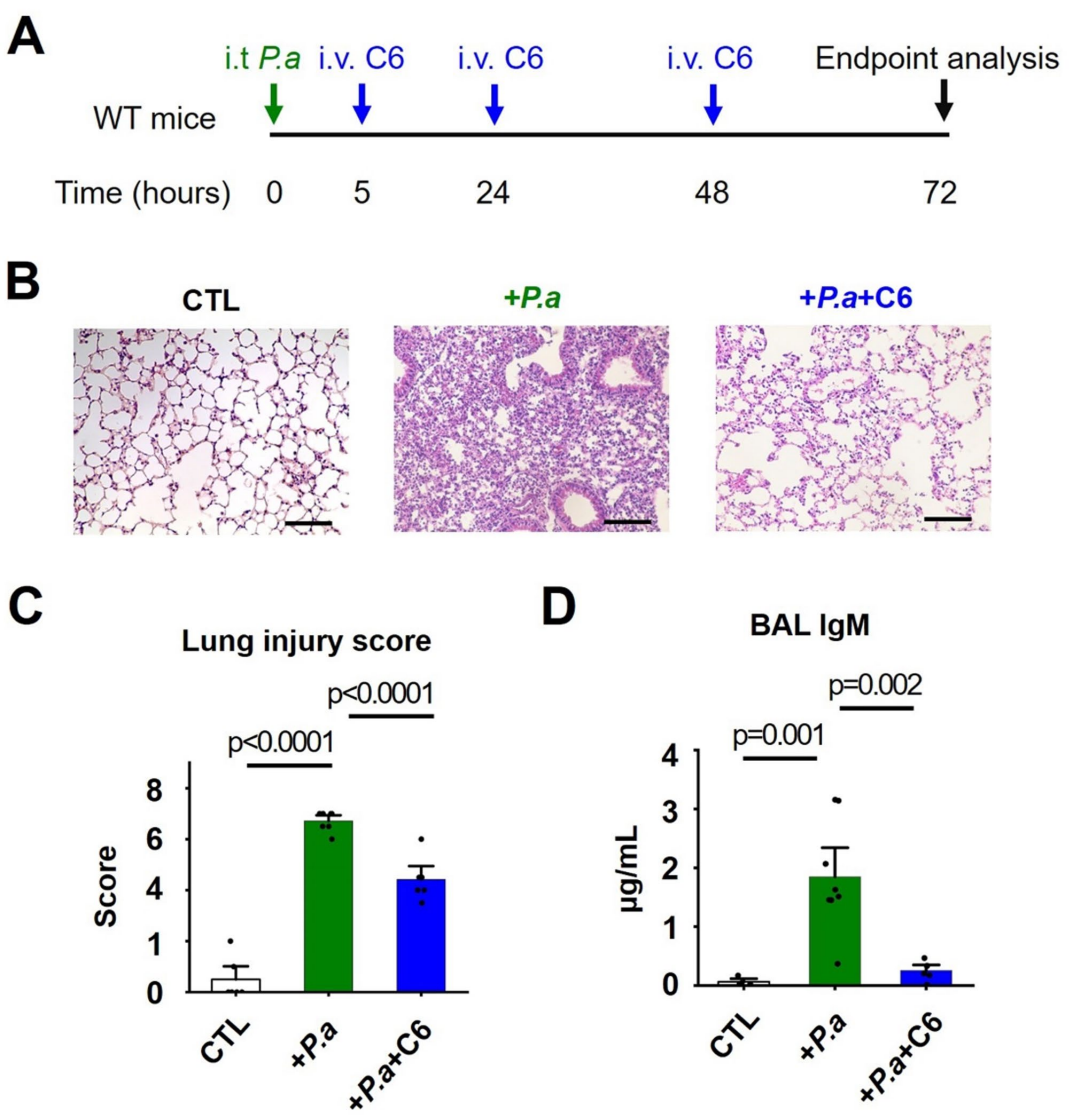
C6 peptide ameliorates P. aeruginosa-induced ALI. Experiments were conducted using WT C57BL/6 mice (age- and sex-matched, *n* = 4–8). C6 (4 mg/kg) was intravenously (i.v.) injected via the retro-orbital vein and histological lung injury scoring was performed 72 h post intratracheal (i.t) instillation of P. aeruginosa. Values are mean ± SEM. Individual dots on bars represent individual mice. **A** Delayed three-dose C6 i.v. administration protocol to mimic a clinical treatment model. **B** Representative images of H&E-stained lung sections of WT mice exposed to P. aeruginosa for 72 h with or without C6 treatment (scale bars = 200 μm). Mice in C6 groups received once-daily i.v. injections of the C6 peptide for three days, as illustrated in the panel A, the control group received injections of PBS, and P. aeruginosa (P.a) groups received injections of P. aeruginosa (2 × 10^7^ CFU) + vehicle control (DMSO: PBS = 1:20). **C** Summary of cumulative lung injury scores, which were determined by an investigator blinded to the experimental conditions on H&E-stained lung sections as described in Methods. Two sets of slides were prepared and analyzed for each mouse, and samples from three mice were analyzed for each group. **D** Measurements of IgM levels of bronchoalveolar lavage (BAL) fluid from mice subjected to P. aeruginosa-induced ALI without or with C6 treatment.

A schematic representation of our P. aeruginosa-induced ALI model is shown in Fig. 1A. Histological analyses and blinded lung injury scoring of hematoxylin and eosin (H&E) stained lung sections revealed moderate-severe lung tissue damage with interstitial thickening and interstitial neutrophilic infiltration in the P. aeruginosa-infected mice compared to control animals (Fig. 1B and C). This level of lung injury allowed for meaningful therapeutic intervention with our C6 peptide.

Immunoglobulin M (IgM) is the first antibody to appear when the body is exposed to bacterial infection. IgM is produced by B cells in the blood compartment and excluded from the healthy alveolar airspace. Thus, the detection of IgM in the alveolar compartment reflects alveolar-capillary barrier breakdown [17]. We observed significant accumulation of IgM in BAL fluid, which demonstrates barrier disruption and increased alveolar-capillary permeability in the P. aeruginosa-infected mice (Fig. 1D).

To mimic a clinically relevant treatment regimen, three doses of C6 (4 mg/kg) were administered i.v. to mice 5, 24, and 48 h after i.t. P. aeruginosa administration (Fig. 1A). We initiated C6 treatment 5 h post-infection and considered it an early therapeutic intervention because, at this time point, mice exhibited a pronounced and pathological decline in body temperature (Fig. S1), accompanied by reduced activity. This C6 dosage was selected based on its demonstrated adequacy to protect mice from LPS-induced ALI, without further benefit from higher dosing [8]. Moreover, pharmacokinetic data for analogous peptides, such as the similarly sized toxin ShK, indicate that relatively high systemic doses are necessary to achieve low-micromolar tissue concentrations [18, 19].

Histological analysis and blinded lung injury scoring of H&E-stained lung sections revealed that the administration of C6 reduced tissue damage (Fig. 1B and C). Further, C6 significantly decreased IgM levels in BAL fluid, suggesting improved integrity of the alveolar-capillary barrier (Fig. 1D).

### C6 reduces neutrophil infiltration, ROS production and elevation of intracellular Ca^2+^ in the lungs of P. aeruginosa-infected mice

**Fig. 2:**
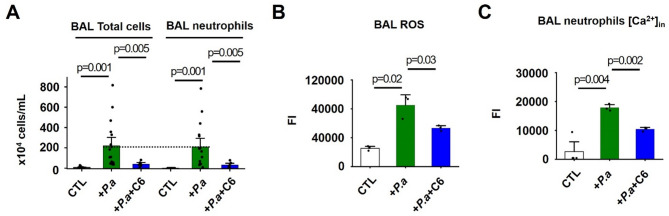
C6 reduces inflammatory response in P. aeruginosa-infected mice. Total cell and neutrophil counts, ROS and intracellular Ca^2+^ ([Ca^2+^]_in_) levels in BAL fluid of mice (*n* = 3–15) were determined as described in Methods. P. aeruginosa and C6 (4 mg/kg) were administered as described in Fig. 1. Control groups received injections of PBS. Values are expressed as mean ± SEM. Individual dots on bars represent individual mice. **A** BAL total cell and neutrophil count for mice subjected to P. aeruginosa-induced ALI without or with C6 treatment. P. aeruginosa infection increased BAL fluid total cell and neutrophil counts, which were reduced by ~ 86% and ~ 82% with C6 administration. **B** ROS production from BAL cells of mice subjected to P. aeruginosa-induced ALI without or with C6 treatment. ROS production by BAL cells was elevated by P. aeruginosa infection, and C6 administration suppressed the rise in ROS by 54%. **C** [Ca^2+^]_in_ of BAL cells of mice subjected to P. aeruginosa-induced ALI without or with C6 treatment. P. aeruginosa infection increased [Ca^2+^]_in_ in BAL cells and C6 administration decreased the elevation by 49%

In our model, the large majority of cells recruited to the lungs upon P. aeruginosa infection were neutrophils (>95% of total cells in BAL fluid), constituting an ~ 8,000-fold increase compared to healthy mouse lungs (Fig. 2A). Such neutrophilic predominance is also observed in patients with bacterial pneumonia [20]. Consistent with the histological lung injury scoring (Fig. 1B and C), administration of C6 reduced the neutrophil cell count in BAL fluid by ~ 86% and the total cell count by ~ 82% (Fig. 2A). Furthermore, ROS production by BAL cells was elevated by 3.3-fold in P. aeruginosa-infected mice compared to controls, and i.v. C6 administration suppressed the P. aeruginosa-induced rise in ROS by 54% (Fig. 2B). The intracellular Ca^2+^ concentration ([Ca^2+^]_in_) in neutrophils regulates a broad spectrum of immune responses, including migration, ROS production, phagocytosis, and degranulation [21]. We found that i.t. P. aeruginosa infection of mice increased the [Ca^2+^]_in_ in BAL cells by ~ 7-fold and i.v. C6 treatment of mice decreased this [Ca^2+^]_in_ elevation by 49% (Fig. 2C).

### C6 reduces proinflammatory cytokines in the BAL fluid of P. aeruginosa-infected mice

During bacterial pneumonia, hyperactive immune cells produce an excessive amount of proinflammatory cytokines, thereby exacerbating pulmonary inflammation [3, 6]. We studied the effect of C6 on the levels of 32 inflammatory cytokines in BAL fluid from P. aeruginosa-infected mice using Luminex’s xMAP^®^ Immunoassay. In response to P. aeruginosa administration, 18 of the 32 cytokines were significantly increased (Table 1). Among these 18 cytokines, C6 significantly suppressed nine, including Interleukin-6 (IL-6), Interferon gamma-induced protein-10 (IP-10), Chemokine CC-motif ligand 2 (CCL2), Macrophage Inflammatory Protein-1 alpha (MIP-1α), Granulocyte Colony-Stimulating factor (G-CSF), Granulocyte Macrophage-Colony Stimulating Factor (GM-CSF), Eosinophil chemotactic protein (Eotaxin), Leukemia Inhibitory Factor (LIF), and Keratinocytes-derived Chemokine (KC, or Interleukin-8). Notably, IL-6, CCL2, GM-CSF, and KC/IL-8 have been identified as key biomarkers of ALI and ARDS, owing to their critical roles in the pathogenesis of pulmonary inflammation, endothelial and epithelial injury, and immune cell recruitment [3]. Three other cytokines, Interferon gamma (IFN-γ), Interleukin-1β (IL-1β), and Macrophage Inflammatory Protein-1β (MIP-1β) were reduced ~ 67%, 87% and 76% by C6, respectively, although the decreases were slightly shy of rejecting the null hypothesis (p = ~ 0.06, 0.07 and 0.07, respectively) (Table 1). We validated these Luminex’s xMAP^®^ Immunoassay findings by quantifying five cytokines individually using conventional ELISA, confirming that C6 reduced IL-6, CCL2, MIP-1α, and IL-1β levels, while the decrease in the level of IFN-γ again reached a p value of 0.07 (Fig. 3).

**Table 1:** Cytokine concentrations in BAL fluid quantified using luminex’s xMAP^®^ Immunoassay. Experiments were conducted using WT C57BL/6 mice (age- and sex-matched, *n* = 5–12). Control groups (CTL) received injections of PBS, and P. aeruginosa (P.a) groups received injections of P. aeruginosa (2 × 10^7^ CFU) + vehicle control (DMSO: PBS = 1:20). C6 (4 mg/kg) was intravenously (i.v.) injected via the retro-orbital vein, and the BAL fluid was collected 72 h post intratracheal (i.t) instillation of P. aeruginosa. The concentration values (pg/mL) are mean ± SEM. The Inhibition shows the percentage of decrease in cytokine levels after C6 treatment

#	Cytokines	CTL	*P*.a	*p* value *P*.a/CTL	*P*.a + C6	*p* value *P*.a/*P*.a + C6	Inhibition
Increase with *P*.a and significant suppression by C6							
1	IL-6	1.3 ± 0.5	1451 ± 444	0.0075	372 ± 171	0.0402	74%
2	IP-10	12.2 ± 2.9	1448 ± 162	0.0001	812 ± 142	0.0110	44%
3	CCL2	2.9 ± 0.3	254 ± 88	0.0161	18.5 ± 6.5	0.0222	94%
4	MIP-1α	3.1 ± 0.3	96 ± 30	0.0099	26.8 ± 6.9	0.0427	75%
5	G-CSF	1.6 ± 0.7	1269 ± 381	0.0068	295 ± 100	0.0290	77%
6	GM-CSF	6.0 ± 0.6	13.0 ± 2.2	0.009	7.9 ± 0.8	0.046	73%
7	Eotaxin	2.6 ± 0.6	9.8 ± 1.9	0.0032	4.3 ± 0.6	0.0167	76%
8	LIF	0.9 ± 0.4	82 ± 23	0.0048	15.4 ± 4.9	0.0153	82%
9	KC/IL-8	2.3 ± 0.9	31.8 ± 6.5	0.0008	14.5 ± 3.6	0.0341	59%
Increase with P.a and not statistically-significant suppression by C6							
10	IFN-γ	0.9 ± 0.3	51 ± 11	0.001	17 ± 12	0.062	67%
11	IL-1β	2.8 ± 0.1	13.2 ± 4.4	0.039	4.2 ± 1.3	0.073	87%
12	MIP-1β	5.3 ± 0.4	145 ± 52	0.022	39.2 ± 11.9	0.073	76%
13	TNF-α	1.5 ± 0.3	23.4 ± 6.4	0.006	10.1 ± 3.0	0.078	61%
14	IL-1α	3.1 ± 0.5	38 ± 12	0.017	14.8 ± 4.0	0.101	66%
15	IL-9	25.9 ± 3.9	51.4 ± 6.1	0.002	37.0 ± 6.2	0.123	56%
16	MIG	3.2 ± 0.9	7452 ± 1976	0.003	3665 ± 2355	0.247	51%
17	IL-12 (p70)	1.7 ± 0.3	2.8 ± 0.3	0.015	2.4 ± 0.6	0.569	37%
18	RANTES	0.6 ± 0.3	30.7 ± 6.6	0.001	28 ± 11	0.832	9%
No significant response to P.a stimulation							
19	IL-2	0.7 ± 0.4	0.42 ± 0.04	0.37	0.38 ± 0.04	0.56	N/A
20	IL-3	0.9 ± 0.4	0.40 ± 0.03	0.28	0.40 ± 0.06	0.94	N/A
21	IL-4	0.8 ± 0.5	0.32 ± 0.03	0.35	0.20 ± 0.04	0.06	N/A
22	IL-5	0.8 ± 0.3	0.63 ± 0.07	0.59	0.50 ± 0.00	0.10	N/A
23	IL-7	0.9 ± 0.4	0.53 ± 0.05	0.37	0.47 ± 0.14	0.70	N/A
24	IL-10	1.9 ± 0.5	2.13 ± 0.56	0.72	1.29 ± 0.27	0.19	N/A
25	IL-12 (p40)	2.8 ± 0.4	5.01 ± 1.06	0.08	6.42 ± 1.30	0.42	N/A
26	IL-13	4.7 ± 1.2	3.81 ± 0.27	0.49	3.68 ± 0.22	0.69	N/A
27	IL-15	3.6 ± 0.7	3.27 ± 0.23	0.62	2.54 ± 0.66	0.34	N/A
28	IL-17	0.9 ± 0.6	7.97 ± 3.31	0.06	6.39 ± 3.14	0.73	N/A
29	LIX	11.5 ± 1.1	17.1 ± 3.1	0.11	10.6 ± 1.8	0.09	N/A
30	M-CSF	2.0 ± 0.3	12.2 ± 5.5	0.09	2.05 ± 0.41	0.09	N/A
31	MIP-2	11.8 ± 1.3	154 ± 79	0.10	24.1 ± 4.9	0.13	N/A
32	VEGF	3.5 ± 0.7	6.78 ± 2.05	0.15	3.81 ± 1.55	0.27	N/A

**Fig. 3:**
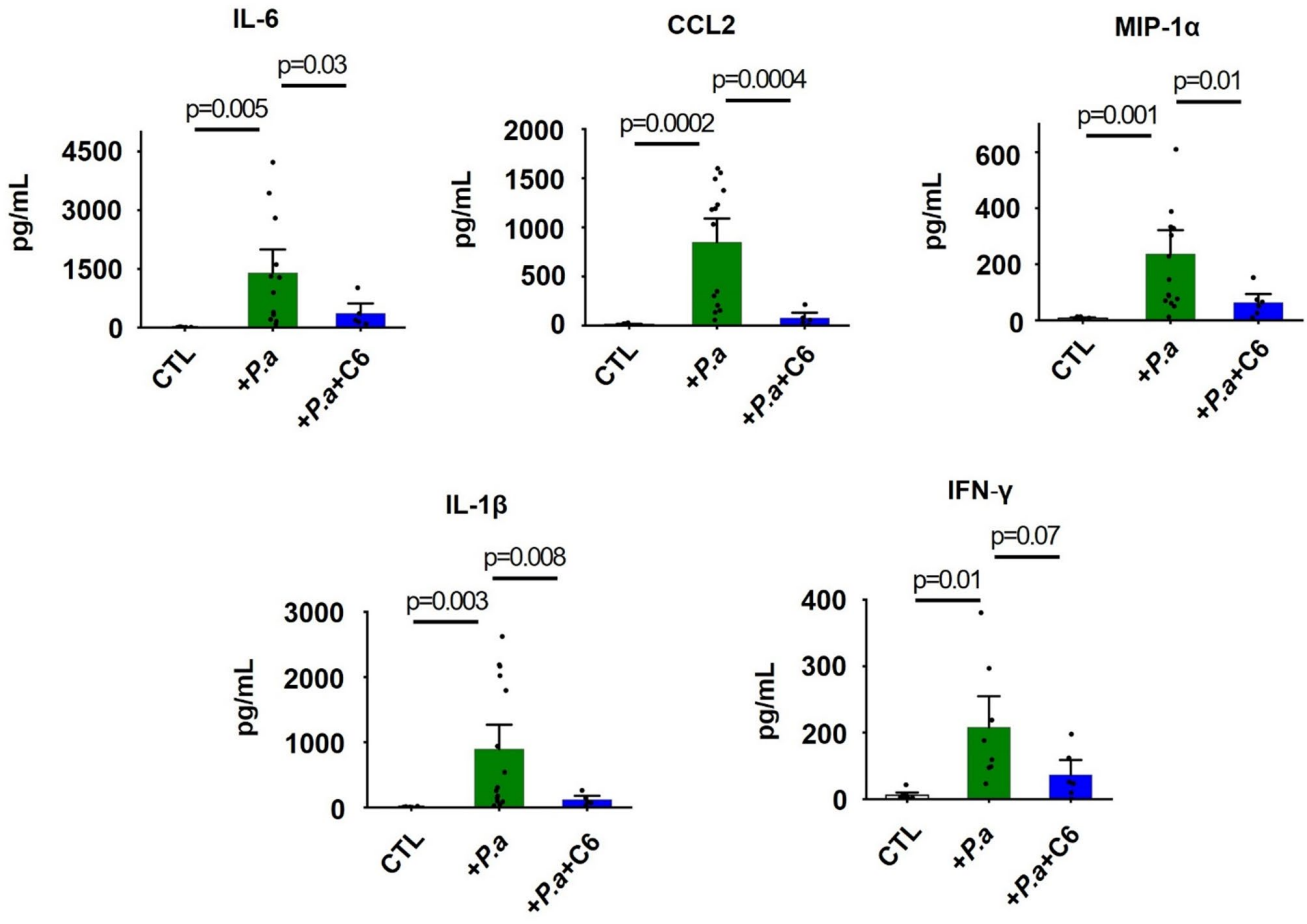
Effects of C6 on BAL fluid cytokine concentrations. Inflammatory cytokine concentrations were quantified in BAL fluid using ELISA. C6 (4 mg/kg) treatment significantly decreased IL-6, CCL2, MIP-1α, and IL-1β concentrations, but not IFN-γ (*p* = 0.07), compared to groups receiving P. aeruginosa. Control groups received injections of PBS. Values are expressed as mean ± SEM; *n* = 5–15 mice. Individual dots on bars represent individual mice

### C6 alters gene expression in BAL neutrophils

**Fig. 4:**
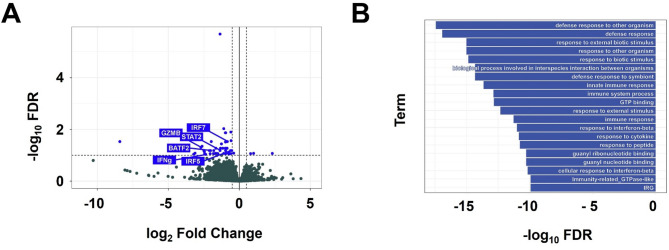
RNA sequencing analysis reveals the impact of C6 on the transcriptome of BAL neutrophils. Experiments were conducted using WT C57BL/6 mice. The P. aeruginosa group received i.t. instillation of P. aeruginosa along with vehicle control. C6 (4 mg/kg) was administered *i.v.* via the retro-orbital vein. BAL neutrophils were isolated 72 h after P. aeruginosa instillation. mRNA isolation, library construction, and sequencing were performed according to the procedures outlined in Methods. *n* = 4 mice in each group. **A** The volcano plot has blue points representing downregulated or upregulated differentially expressed genes, and dark green points representing non-differentially expressed genes. **B** Gene Ontology enrichment analysis of differentially expressed genes

To further elucidate the mechanism by which C6 protects the lungs of P. aeruginosa-infected mice, we performed transcriptomic analysis using mRNA isolated from BAL neutrophils. Differential gene expression analysis was conducted to identify genes that were upregulated or downregulated in response to C6 treatment, based on the following criteria: (1) exclusion of low-expression genes (defined as fewer than 10 counts in at least 5 samples); (2) a log_2_ fold change greater than 0.5 or less than − 0.5; and (3) a false discovery rate (FDR)-adjusted p-value below 0.1. Using these thresholds, we identified 54 differentially expressed genes (Fig. 4A), including 3 upregulated and 51 downregulated genes (Table S1). Gene Ontology (GO) analysis revealed that the principal functional categories significantly affected in BAL neutrophils following C6 treatment included defense response, innate immune response, response to interferon signaling and immunity-related GTPase-like, among others (Fig. 4B).

Among the 51 genes downregulated by C6 treatment, two, *Interferon Regulatory Factor 7* (IRF7) and *Granzyme B* (GZMB), have been reported to promote neutrophil migration [22, 23]. Additionally, two genes, Signal Transducer and Activator of Transcription 2 (STAT2) and Interferon Regulatory Factor 5 (IRF5), have been associated with the regulation of ROS production [24, 25]. Five genes, IRF7, STAT2, *Basic Leucine Zipper ATF-Like Transcription Factor 2* (BATF2), IRF5, and IFNG [22, 24–27], have been reported to be essential for robust cytokine release. C6 treatment reduced the expression levels of these genes by 24% to 84% (Table 2), indicating a broad suppressive effect on inflammatory pathways.

**Table 2:** List of genes expressed in BAL neutrophils that are downregulated by C6 treatment and influence the neutrophil inflammatory response. Experiments were conducted using WT C57BL/6 mice. The P. aeruginosa group received i.t. Instillation of P. aeruginosa along with vehicle control. C6 (4 mg/kg) was administered *i.v.* Via the retro-orbital vein. BAL neutrophils were isolated 72 h after P. aeruginosa Instillation, as described in the Methods. mRNA isolation, library construction, and sequencing were performed according to the procedures outlined in Methods. All differentially expressed genes are presented in Table S1. False discovery rate (FDR), an adjusted p-value threshold of < 0.1 indicates that approximately 10% of the genes identified as “significant” may represent false positives. Gene expression levels were determined based on the basemean value, where values between 100 and 1000 indicate moderate to high expression, and values greater than 1000 correspond to high expression levels. The percentage of downregulation by C6 treatment was calculated based on the log₂ fold change of the genes. The roles of genes in neutrophil inflammatory responses were annotated based on published literature. A filled circle (●) indicates that the gene is involved in the corresponding neutrophil function

	Gene	Expression level	FDR	Downregulation by C6	Neutrophils		
					Migration	ROS	Cytokines
**1**	IRF7	High	0.03	45%	●		●
**2**	GZMB	Moderate to high	0.04	84%	●		
**3**	STAT2	High	0.05	50%		●	●
**4**	BATF2	Moderate to high	0.07	71%			●
**5**	IRF5	High	0.08	24%		●	●
**6**	IFNG	Moderate to high	0.09	81%			●

Conversely, three genes were upregulated following C6 treatment: Macrophage Receptor with Collagenous Structure (MARCO), Mitogen-Activated Protein Kinase RAS (MRAS), and Methylenetetrahydrofolate Dehydrogenase [NADP⁺ Dependent] 2-Like (MTHFD2L) that influence neutrophil function by modulating pathogen recognition, intracellular signaling, and metabolic adaptation (**Table ****S1**) [28–30]. These genes were upregulated ~ 2-, 5-, and 2-fold, respectively.

### C6 peptide inhibits human neutrophil migration

To further study the inhibitory effects of C6 on neutrophil function, we performed a trans-well chemotaxis assay to quantify migration using freshly isolated human neutrophils. Neutrophils isolated from healthy donors were treated with C6 or control blockers and seeded in the upper chamber in serum-free medium, and two well-established neutrophil chemoattractants, the bacterial endotoxin LPS or the endogenous proinflammatory cytokine IL-1β [31, 32], were added individually to the lower chamber to stimulate migration across the permeable polyester membrane support containing 3.0 μm pores. LPS and IL-1β were selected as chemoattractants to replicate the contributions of microbial products and host-derived inflammatory mediators that drive neutrophil recruitment during P. aeruginosa infection.

**Fig. 5:**
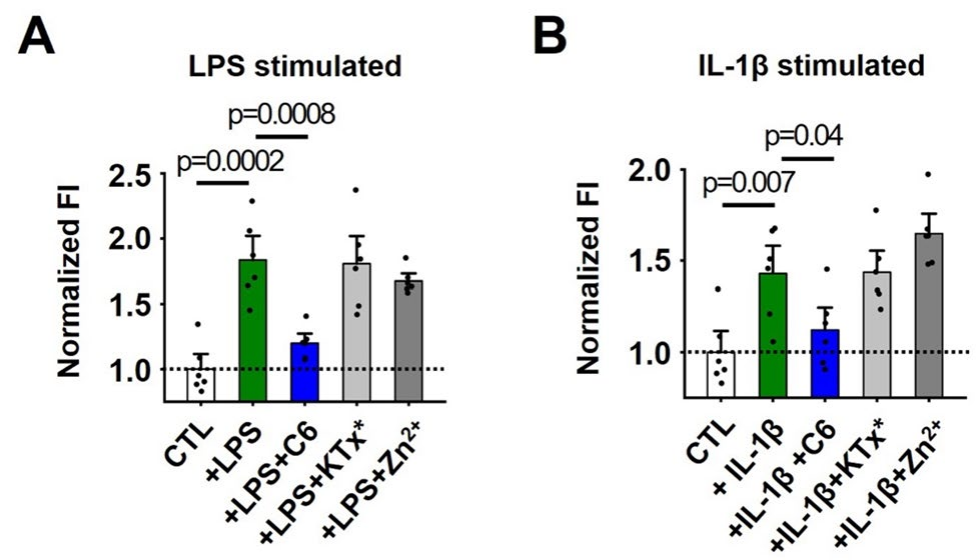
C6 inhibits human neutrophil migration. Human neutrophils were isolated from the peripheral blood of healthy volunteers. The migration of neutrophils was studied using trans-well migration assays as described in the Methods. C6 (2 µM), negative control peptide KTx* (2 µM), and non-specific blocker Zn^2+^ (2 µM) were incubated with neutrophils (5 × 10^5^ cells/well). LPS (25 µg/mL) and IL-1β (0.5 µg/mL) were used to stimulate neutrophil migration. The rate of migration was determined 24 h after the beginning of the assay. Values are mean ± SEM; *n* = 6 for each condition. **A** LPS-induced neutrophil migration without or with C6. **B** IL-1β-induced neutrophil migration without or with C6

After 24 h, both LPS and IL-1β increased the number of neutrophils that migrated through the pores into the lower chamber when compared to the control group, as indicated by an increase in fluorescence (Fig. 5). This migration was inhibited by 2 µM C6 by 76% and 72% for the LPS and IL-1β groups, respectively (Fig. 5). In contrast, a control peptide KTx* that does not inhibit Hv1 channels [33] and Zn^2+^, a low affinity and non-specific blocker of Hv1 channels [34], had negligible effects on the migration of human neutrophils at the same concentration (Fig. 5).

### C6 suppresses the production of pro-migratory, proinflammatory responses of human neutrophils induced by P. aeruginosa

**Fig. 6:**
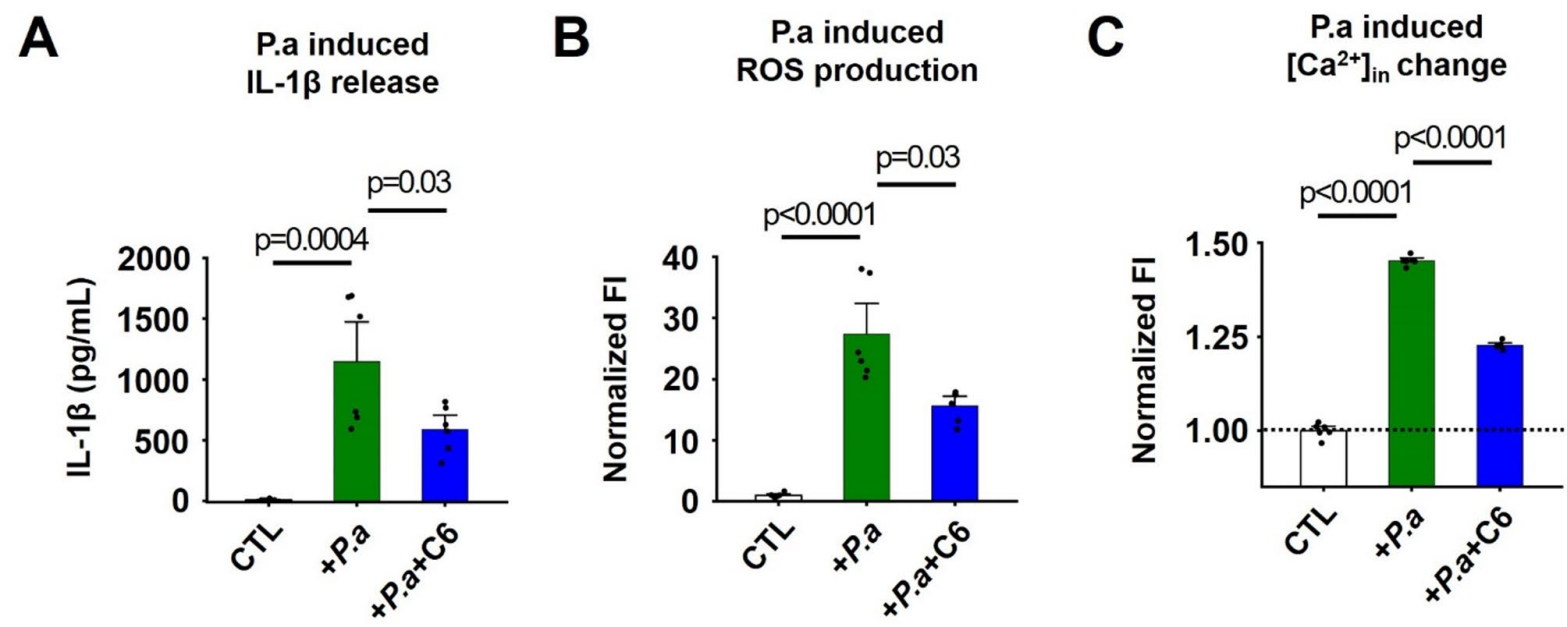
C6 suppresses P. aeruginosa-induced inflammatory responses in human neutrophils Human neutrophils were isolated from the peripheral blood of healthy volunteers and studied using microplate fluorometer (Methods). Values are expressed as mean ± SEM; *n* = 6 for each condition. **A** Effect of P. aeruginosa (3 × 10^7^ CFU) without or with C6 peptide (2 µM) on IL-1β release by human neutrophils (1 × 10^6^ cells). The concentration of IL-1β was quantified using ELISA. **B** Effect of P. aeruginosa (6 × 10^6^ CFU) without or with C6 peptide (2 µM) on ROS release by human neutrophils (2 × 10^5^ cells). ROS was measured using Luminol. ROS release 5 min after P. aeruginosa stimulation was plotted. Values are normalized to mean ROS release by neutrophils without P. aeruginosa stimulation (CTL). **C** Effect of P. aeruginosa (3 × 10^6^ CFU) without or with C6 peptide (2 µM) on intracellular Ca^2+^ level of neutrophils (1 × 10^5^ cells) 5 min after P. aeruginosa stimulation. [Ca^2+^]_in_ was measured using Fluo-3. Values are normalized to mean [Ca^2+^]_in_ of neutrophils without P. aeruginosa stimulation (CTL)

Proinflammatory cytokines play an important role in the development and disease progression of bacterial pneumonia. For example, the serum level of IL-1β during the early stages of pneumonia correlates with disease severity [35]. Human neutrophils produce and release IL-1β during infection, and IL-1β induces directional migration of neutrophils to inflamed sites [36]. Here, we observed that P. aeruginosa-induced IL-1β release by human neutrophils using ELISA is ~ 144-fold higher than controls and this increase was suppressed by 49% with C6 treatment (Fig. 6A).

Human neutrophils undergo a respiratory burst to produce ROS, a principal effector mechanism for bacterial killing [7]. During the respiratory burst, the NADPH oxidase 2 (NOX2) transfers electrons across the cell membrane, resulting in membrane depolarization and cytoplasmic acidification that suppresses ROS production [37]. To sustain NOX2 activity and ROS production, we have shown that proton efflux via human Hv1 is required to maintain a physiological intracellular pH and cell membrane potential [14, 38]. Moreover, ROS regulates both basal and directional migration of neutrophils [39]. Here, we used a Luminol amplified chemiluminescence assay to assess ROS production by neutrophils [14], observing that P. aeruginosa stimulation increased ROS production by ~ 27-fold, and C6 suppressed the increase of ROS by 45% (Fig. 6B).

Changes in [Ca^2+^]_in_ control neutrophil activation and function [21] and transient increases are proposed to be required for the migration of human neutrophils [40]. Here, we found that P. aeruginosa stimulation of human neutrophils increased [Ca^2+^]_in_ ~1.5-fold and that C6 suppressed this increase by 50% (Fig. 6C).

## Discussion

The development of novel therapeutic approaches against ALI/ARDS is urgently needed since current therapies are largely symptomatic and often promote further lung injury (e.g., hyperoxia, mechanical ventilation) or cause significant side effects (e.g., antibiotics). Defined as a non-cardiogenic lung disease with acute onset and characterized by diffuse bilateral pulmonary infiltrates due to alveolar cellular inflammation and alveolar-capillary barrier dysfunction resulting in lung edema with protein-rich fluid that impairs arterial oxygenation [3], ALI/ARDS is seen in ~ 190,000 patients in the United States each year, and associated with ~ 74,500 deaths [4]. In almost half of the cases, a primary pulmonary source of injury is detected, most commonly an infection. Pulmonary infections with P. aeruginosa are particularly important as they are associated with high morbidity and mortality rates in hospitalized and immunocompromised patients, notably those with cystic fibrosis, bronchiectasis, neutropenia, cancer, burns, AIDS, and organ transplants. Indeed, in intensive care units, P. aeruginosa is detected in almost all patients with long-term ventilation [41].

Here, we focus on neutrophils, as they contribute to both protection from bacterial infections and the development of ALI/ARDS injury [4, 5], and present the predominant inflammatory cell type recruited to the lungs during pneumonia. In our model, we demonstrate that inhibition of the Hv1 channel can suppress the injurious sequelae of live P. aeruginosa pulmonary infection and neutrophil activation. We identified the direct inhibition of alveolar-capillary barrier damage by suppressing neutrophil migration into the lungs and reducing excess local production of inflammatory mediators as the primary mechanism of protection by C6 peptide.

### A mechanistic view of C6 protection by suppression of neutrophil migration

In ALI/ARDS patients, higher neutrophil counts are a predictor of poor outcomes [42]. As first responders to pulmonary inflammation, neutrophils contribute to tissue damage primarily through the release of ROS, proteases, and the amplification of cytokine production, thereby exacerbating the inflammatory cascade and promoting cytokine storm. Strategies to diminish neutrophils in lung tissue, including decreasing neutrophil recruitment and impairing neutrophil inflammatory response, have been predicted to attenuate lung injury [43]. Here, we demonstrate that our C6 peptide accomplishes these intimately related goals by significantly suppressing, but not eliminating, neutrophil migration into the lungs (Figs. 1B, 2A and 5), decreasing ROS, pro-migratory cytokines, and [Ca^2+^]_in_ (Figs. 2, 3 and 6A), and demonstrating that this protects mice from P. aeruginosa-induced lung injury in a clinically relevant live bacterial infection model (Fig. 1).

The mechanism of inhibition of neutrophil migration results from at least four identified effects of C6 blockade of Hv1. First, C6 decreases the levels of six proinflammatory cytokines/chemokines in BAL fluid from P. aeruginosa-infected mice that drive neutrophil migration (Table 1 and Fig. 3). Thus, CCL2 and MIP-1α are reported to mediate firm adherence and subsequent transmigration of neutrophils [44]; G-CSF and GM-CSF are growth factors that stimulate neutrophils to migrate across endothelial cells to sites of infection and inflammation [45]; KC/IL-8 appears to be a critical chemokine in neutrophil recruitment during lung infections [46] and BAL KC/IL-8 levels correlate strongly with neutrophil counts, disease severity, and poor clinical outcomes [47, 48]. In our study, C6 administration reduced KC/IL-8 levels in mouse BAL fluid by 59% (*p* = 0.03; Table 1), underscoring its anti-inflammatory and protective effects in this model; IL-1β causes dose- and time-dependent neutrophil migration in different animal models [49, 50], antibodies against IL-1β attenuate neutrophil migration [51], and IL-1β stimulates directional neutrophil migration [36]. We found that the level of IL-1β in mouse BAL fluid was decreased by C6 administration (*p* = 0.008, Fig. 3) and confirmed that treating human neutrophils with C6 decreased IL-1β release (*p* = 0.03, Fig. 6A).

Second, C6 treatment downregulated the expression of genes in BAL fluid neutrophils associated with migration (Table 2). For instance, IRF7 plays a critical role in type I interferon production and enhances neutrophil recruitment to tissues [26]. Attenuation of IRF7 expression has been shown to reduce neutrophil infiltration into the BAL fluid following viral infection [52]. Additionally, neutrophils upregulate GZMB upon infection, leading to the secretion of granzyme B, which contributes to extracellular matrix remodeling and the disruption of endothelial junctions, thereby facilitating neutrophil extravasation and tissue migration [22]. A reduction in GZMB expression may impair these processes.

Third, C6 suppresses ROS production in P. aeruginosa-infected mice (Fig. 2), and ROS regulates neutrophil migration [31]. Thus, depletion of ROS interferes with the directed movement of neutrophils up a chemoattractant concentration gradient [53]. Previously, we showed that C6 suppresses 71% of ROS production by neutrophils as a central protective effect of Hv1 inhibition [8, 14]; this is due to blockade of proton efflux, decreasing intracellular pH, and depolarization of the cell membrane, two effects that inhibit the activity of NOX2 and preclude sustained ROS production [14]. In addition to NOX2, the primary source of ROS in neutrophils, C6 treatment also downregulated the expression of STAT2 and IRF5 (Table 2), both of which are essential for ROS production in neutrophils. Mice deficient in either STAT2 or IRF5 exhibit significantly impaired ROS generation [24, 25].

Fourth, C6 suppresses the P. aeruginosa-induced elevation of [Ca^2+^]_in_ in neutrophils (Fig. 2C). It is known that Ca^2+^ influx triggers cytoskeletal rearrangement and polarization, which is necessary for the migration of neutrophils [54] and transient increases in [Ca^2+^]_in_ are linked to directional migration [40]. One effect of C6-induced depolarization of the neutrophil plasma membrane could be to hinder Ca^2+^ influx by decreasing the electrochemical driving force and decreasing the open probability of voltage-gated Ca^2+^ channels [54].

### LPS-induced vs. P. aeruginosa-induced ALI

Previously, in a proof-of-concept study, we used a simple LPS-induced lung injury model to assess whether C6 could convey anti-inflammatory effects and found, for the first time, that C6 treatment of mice decreases neutrophil influx into the lungs [8]. Whether these encouraging results obtained with the relatively artificial LPS model can be translated into a clinically more relevant model of live bacterial infection, and what mechanisms convey potential C6 protection, remained unknown and was thus the focus of the current study. We infected mice i.t. with P. aeruginosa, followed by three i.v. doses of C6 at 5, 24, and 48 h post-infection and measured clinically-relevant inflammatory endpoints at 72 h. Compared to LPS, P. aeruginosa infection elicited a markedly more robust inflammatory response, including an ~ 8,700-fold increase in neutrophil infiltration relative to baseline, which was suppressed by ~ 86% following C6 treatment, even more than the ~ 60% reduction observed in the LPS model (Table 3). In addition to neutrophil accumulation, P. aeruginosa induced stronger cytokine responses in the lungs (Table 3). With the exception of IL-1β, the increases of IL-6, CCL2, IP-10, MIP-1α, and TNF-α were all greater in the P. aeruginosa model than in LPS-induced lungs. Despite this intensified cytokine “storm”, C6 exhibited superior anti-inflammatory efficacy in the P. aeruginosa model, as evidenced by larger percentage decreases across all cytokines when compared to the LPS model (Table 3). The pattern was reversed for ROS suppression: C6 reduced LPS-induced ROS levels by 71%, but achieved only a 54% reduction in the P. aeruginosa model. These findings demonstrate that C6 retains its protective and anti-inflammatory activity in the context of a complex, clinically relevant model of live bacterial infection. The data suggest that C6 exerts its protective effects, at least in part, by inhibiting neutrophil recruitment and activation, even in the presence of an intensified inflammatory milieu.

**Table 3:** Comparison of C6 effects in P. aeruginosa- and LPS-induced ALI models. This table compares the effects of C6 treatment on neutrophil infiltration, cytokine production, and ROS levels in BAL fluid from mice subjected to either P. aeruginosa infection or LPS-induced ALI. For each model, the fold increase (mean ± SEM) in neutrophil counts, cytokine concentrations, and ROS levels relative to vehicle-treated controls is reported. Additionally, the percentage decrease in these parameters following C6 treatment is provided. Data for the P. aeruginosa and P. aeruginosa + C6 groups were obtained from Fig. 2A; table 1, and Fig. 2B, whereas data for the LPS and LPS + C6 groups were extracted from our previously published work [8]

BAL	*P*. aeruginosa (fold increase)	*P*. aeruginosa + C6 (% decrease)	LPS (fold increase)	LPS + C6 (% decrease)
Neutrophils count	8700 ± 2300	86%	1400 ± 290	60%
IL-6	1100 ± 340	74%	415 ± 40	49%
CCL2	88 ± 30	94%	52 ± 13	66%
IP-10	119 ± 13	44%	7 ± 0.4	34%
IL-1β	5 ± 0.2	87%	84 ± 14	51%
MIP-1α	31 ± 10	75%	19 ± 2	34%
TNF-α	16 ± 4	61%	14 ± 3	42%
ROS	3 ± 0.4	54%	4 ± 0.4	71%

Furthermore, we conducted new mechanistic investigations, including measurements of intracellular Ca^2+^ dynamics and gene expression profiling for P. aeruginosa-induced ALI. These analyses revealed mechanisms by which C6 modulates neutrophil function. Specifically, Hv1 inhibition was found to alter Ca^2+^ signaling and regulate IFN-related gene expression, thereby linking proton channel activity to Ca^2+^-mediated neutrophil migration and activation. These findings also represent new insights beyond our prior observations of reduced ROS and cytokine production in the innate immune neutrophil response to chemical stimulation by LPS, formylated bacterial peptide fMLF and phorbol myristate acetate (PMA) [8, 9, 14].

To validate the anti-inflammatory effects of C6 observed in mice, we conducted experiments using human neutrophils, including migration and Ca^2+^ influx assays and cytokine release assays. These experiments demonstrated the translational relevance of our findings to human neutrophils and the potential use of C6 as a therapeutic agent.

### Cellular targets of C6 action

Neutrophils are not present in healthy alveoli [3]. During infection, alveolar epithelial cells, endothelial cells, and resident macrophages secrete cytokines, notably, CCL2, MIP-1α, KC/IL-8, G-CSF, GM-CSF and IL-1β that recruit and activate circulating neutrophils to migrate into the alveoli, damaging the alveolar-capillary barrier by releasing ROS and proteases that damage endothelial and epithelial cells, and allowing influx of fluid into the air spaces causing hypoxemia [43]. Thus, neutrophils play a central role in the initiation and progression of ALI/ARDS (4), and their numbers in BAL fluid correlate with disease severity and patient outcomes [20].

Consistent with previous reports, we observe that neutrophil numbers are negligible in healthy mouse lungs and significantly elevated in the BAL fluid of P. aeruginosa-infected mice (Fig. 2A). In addition, the protective effects of C6 peptide against P. aeruginosa infection, i.e. a reduction in BAL fluid neutrophil counts and in pro-migratory and proinflammatory mediator secretion appear to be mediated via inhibition of Hv1 channels in neutrophils. However, Hv1 channels are also expressed at significant levels in alveolar macrophages and epithelial cells [55], suggesting the salutary effects of C6 may also result from its actions on these cell types. In macrophages, Hv1 facilitates the respiratory burst and regulates phagosomal pH. Its inhibition disrupts NOX2-dependent ROS generation and alters intracellular pH homeostasis [56], which can influence downstream inflammatory pathways, including NF-κB activation and inflammasome signaling [57]. Thus, C6 may mitigate macrophage-mediated inflammation by limiting ROS and cytokine production during infection. Hv1 channels in airway epithelial cells help maintain airway surface liquid pH and contribute to mucosal defense [58]. Although their exact role in ALI remains unclear, Hv1 blockade in epithelial cells could modulate local inflammatory responses by affecting redox signaling or cytokine secretion.

### Gene targets of C6 action

Our RNA-seq analysis provides transcriptomic insights into gene expression changes in BAL neutrophils following inhibition of the Hv1 channel. C6 treatment resulted in the significant downregulation of 51 genes and the upregulation of 3 genes. Among the downregulated genes, IRF7 and STAT2 are components of the type I Interferon (IFN) signaling pathway. IRF7 plays a central role in the amplification loop of type I IFN production, while STAT2 is a key mediator of interferon-stimulated gene expression, orchestrating broad anti-infective and immunomodulatory responses. The type I IFN signaling cascade is critical for regulating neutrophil activation, survival, migration, ROS generation, and cytokine secretion [59]. Notably, genetic or pharmacological inhibition of type I IFN signaling has been shown to protect against P. aeruginosa-induced lung injury by reducing neutrophil extracellular trap (NET) formation and bacterial persistence [60]. Thus, the observed downregulation of these genes suggests that C6 treatment attenuates type I IFN-mediated neutrophil inflammatory responses.

Among the few upregulated genes, MARCO, a scavenger receptor, facilitates the clearance of particulate debris and has been shown to suppress early neutrophil-driven inflammation in the lung [28]. MTHFD2L encodes a mitochondrial enzyme involved in one-carbon metabolism. Upregulation of such enzymes can support biosynthesis and promote anti-inflammatory reprogramming of immune cells [29]. MRAS, a member of the Ras family of GTPases, has an undefined role in neutrophils, but Ras signaling broadly regulates neutrophil polarity and survival, and Ras-GAP proteins such as RASAL3 are known to constrain neutrophil hyperactivation [30].

Collectively, these transcriptomic alterations, characterized by downregulation of inflammatory response and type I IFN signaling pathways alongside the upregulation of scavenger and metabolic regulators, suggest that C6 treatment reprograms BAL neutrophils away from a hyper-inflammatory phenotype and toward enhanced phagocytic and immunomodulatory functions. This transcriptional reprogramming likely underpins the anti-inflammatory and tissue-protective effects observed with Hv1 blockade in the P. aeruginosa-induced ALI model.

#### Therapeutic potential of C6 peptide

Despite over a century of intense research efforts and disease modeling, no molecular targets have been identified that improve the outcomes of patients with pneumonia [3, 42]. In the present study, C6-treated mice exhibited no signs of distress or acute toxicity. No abnormal behaviors, weight loss, hypothermia, or mortality were observed compared with vehicle-treated controls during the 72-hour observation period. Consistent with our previous findings in an LPS-induced ALI model, C6 produced no baseline effects in uninjured mice; it did not alter lung compliance, neutrophil counts, or BAL ROS levels in healthy animals [8]. Collectively, these results suggest that selective Hv1 blockade by C6 is well tolerated and not associated with major adverse effects, although further comprehensive off-target and long-term safety evaluations are warranted.

It is also notable that suppression, but not elimination, of neutrophil migration and inflammatory mediator release may offer a fine balance between protecting mice from lung injury and not suppressing the innate immune response so significantly that the animals succumb to an overwhelming bacterial infection since complete neutrophil depletion is reported to cause a fatal defect in pulmonary bacteria clearance [61].

The potential use of C6 peptide as a therapeutic agent is supported by its stability in solution for several months without loss of activity. We attribute this longevity to six cysteines that form three intramolecular disulfide bonds, thereby maintaining a 3D scaffold in C6, similar to that present in natural ICK toxins [9]. One disadvantage of peptides as drugs is that many are subject to rapid renal clearance [62]. Thus, venom-derived peptides similar in size to C6 have a half-life of ~ 1 h in vivo [63]. Conversely, the increasing use of peptide therapeutics has provided modification strategies to prolong their survival [62, 64], which may prove advantageous if required.

Hv1 has been implicated in many biological processes, including white blood cell immune responses [8, 9, 14, 65], sperm capacitation [9, 14, 66], cancer cell proliferation [55, 67], tissue damage after ischemic stroke and spinal cord injury [68, 69], insulin secretion [70] and pain development [71, 72]. The demonstration that C6 suppresses infectious ALI and the specific targeting of Hv1 by C6 without apparent deleterious side effects suggests that both Hv1 as a target and C6 may find utility in treating other inflammatory diseases.

## Supplementary Information

Supplementary Material 1.

Supplementary Material 2.

## Data Availability

The authors confirm that the data supporting the findings of this study are available within the article. Additional data will be made available upon reasonable request.
