# Supplementary figures and images for: C6 peptide blockade of Hv1 channels inhibits neutrophil migration into the lungs to suppress Pseudomonas aeruginosa-induced acute lung injury
Source: Respir Res. 2025 Nov 28;26:339. doi: 10.1186/s12931-025-03409-0 (PMC12661846; doi:10.1186/s12931-025-03409-0)

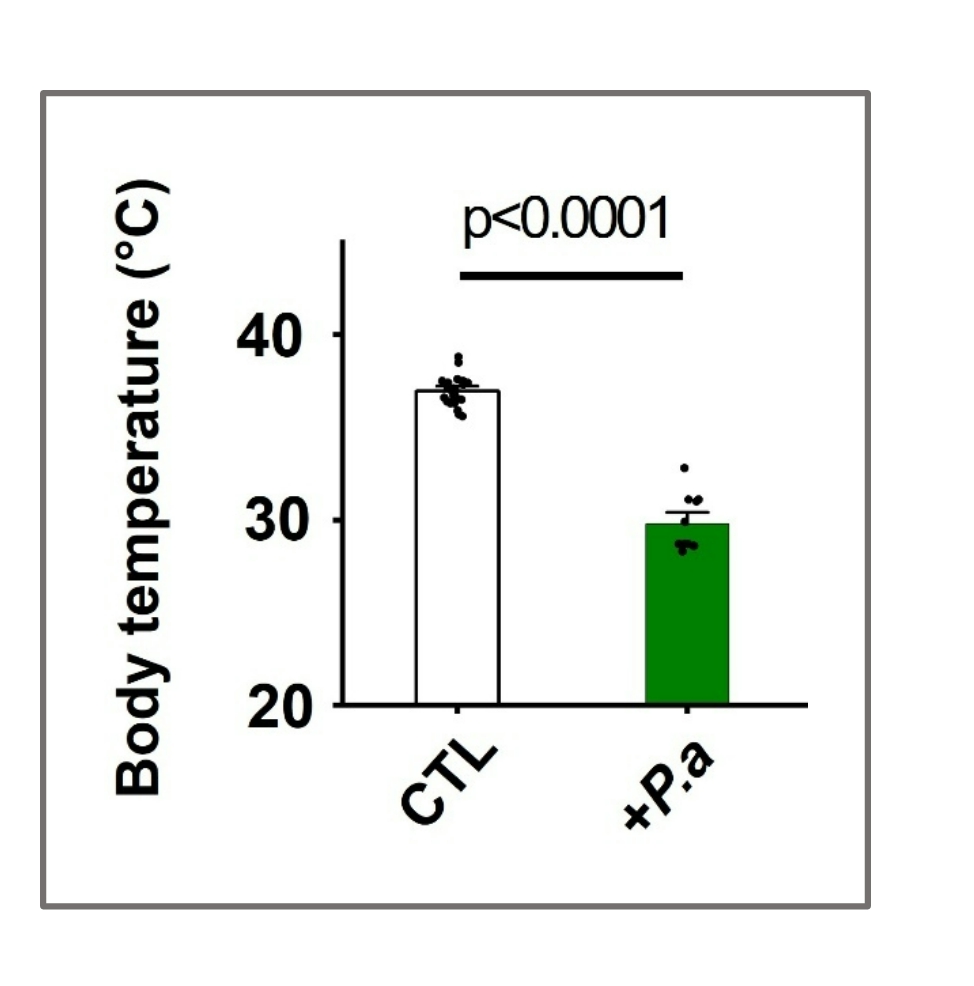

Supplement: Supplementary file 2 — Supplementary Material 2. [file 12931_2025_3409_MOESM2_ESM.jpeg]
